# Virtual Reality Based Cognitive Rehabilitation in Minimally Conscious State: A Case Report with EEG Findings and Systematic Literature Review

**DOI:** 10.3390/brainsci10070414

**Published:** 2020-07-01

**Authors:** Maria Grazia Maggio, Antonino Naro, Gianluca La Rosa, Alice Cambria, Paola Lauria, Luana Billeri, Desiree Latella, Alfredo Manuli, Rocco Salvatore Calabrò

**Affiliations:** Rocco Salvatore Calabrò, IRCCS Centro Neurolesi Bonino Pulejo, via Palermo, SS 113, Ctr. Casazza, 98124 Messina, Italy; mariagrazia.maggio@irccsme.it (M.G.M.); antonino.naro@irccsme.it (A.N.); gianluca.larosa@irccsme.it (G.L.R.); alice.cambria@irccsme.it (A.C.); paola.lauria@irccsme.it (P.L.); luana.billeri@irccsme.it (L.B.); desiree.latella@irccsme.it (D.L.); alfredo.manuli@irccsme.it (A.M.)

**Keywords:** disorders of consciousness, unresponsive wakefulness syndrome, minimally conscious state, virtual reality training, Bts nirvana, EEG

## Abstract

Chronic disorders of consciousness cause a total or partial and fluctuating unawareness of the surrounding environment. Virtual reality (VR) can be useful as a diagnostic and/or a neurorehabilitation tool, and its effects can be monitored by means of both clinical and electroencephalography (EEG) data recording of brain activity. We reported on the case of a 17-year-old patient with a disorder of consciousness (DoC) who was provided with VR training to improve her cognitive-behavioral outcomes, which were assessed using clinical scales (the Coma Recovery Scale-Revised, the Disability Rating Scale, and the Rancho Los Amigos Levels of Cognitive Functioning), as well as EEG recording, during VR training sessions. At the end of the training, significant improvements in both clinical and neurophysiological outcomes were achieved. Then, we carried out a systematic review of the literature to investigate the role of EEG and VR in the management of patients with DoC. A search on PubMed, Web of Science, Scopus, and Google Scholar databases was performed, using the keywords: “disorders of consciousness” and “virtual reality”, or “EEG”. The results of the literature review suggest that neurophysiological data in combination with VR could be useful in evaluating the reactions induced by different paradigms in DoC patients, helping in the differential diagnosis. In conclusion, the EEG plus VR approach used with our patient could be promising to define the most appropriate stimulation protocol, so as to promote a better personalization of the rehabilitation program. However, further clinical trials, as well as meta-analysis of the literature, are needed to be affirmative on the role of VR in patients with DoC.

## 1. Introduction

Unresponsive wakefulness syndrome (UWS) and minimally conscious state (MCS) are chronic disorders of consciousness (DoC) that can occur after a severe brain injury. In such conditions, the patient is totally or partially and variably unaware of and unresponsive to the surrounding environment, respectively. Patients with UWS do not show evidence of intentional or voluntary behavioral responsiveness to visual, auditory, tactile or harmful stimuli. Conversely, patients with MCS show intentional or voluntary behavioral responsiveness to visual and not simple reflexive activity only [1,2,3,4]. The gold standard of DoC diagnosis is currently based on repeated clinical evaluations by using standardized scales, including the Coma Recovery Scale-Revised (CRS-R) [5], which are based on the observation of the patient’s spontaneous and stimulus-induced behaviors and/or on the information of healthcare professionals and family members [6,7]. These procedures can however lead to diagnostic biases that could negatively affect the therapeutic and rehabilitative decisions [6,8]. In fact, interpreting the responses of a patient subjected to sensory stimulation can lead to over- or under-estimating the level of activation, which can lead to a rehabilitation not appropriate to the real residual capacities of the patient. Therefore, advanced assessment tools are mandatory [9,10,11,12]. With this regard, virtual reality (VR) may be of some help in both diagnostic and neurorehabilitation treatment [13,14].

VR refers to computer-simulated environments simulating the sense of presence as in the real world and in a fully controllable manner. VR scenarios are provided through computer screens, stereoscopic displays, wearable glasses, and floor/wall projectors. As a diagnostic tool, VR can be used to manipulate variables that otherwise could not be manipulated in reality, allowing a careful evaluation of the patient, by presenting salient stimuli on a large screen. On the other side, VR allows a multisensory stimulation with augmented feedback, with the possibility of customizing therapeutic sessions and promoting high levels of involvement and activation in neurological patients, including DoC [15,16,17]. Actually, the peculiar characteristics of VR can be set finely in order to address cognitive functions appropriately, thus maximizing the benefits of therapeutic sessions. The rationale of using multisensory stimulation in patients with DoC stems from the fact that sensory deprivation is critically correlated with motor-related disabilities [18,19]. VR is implemented essentially to increase the level of arousal and awareness through stimulating the reticular activating system [20,21] in order to facilitate that environmental inputs entrain awareness networks, even though the degree of disability remains high [22]. However, the results of multisensory stimulation can be discerned from spontaneous recovery (that may occur within the first three months after an injury) difficulty. Therefore, the efficacy of VR-based approaches in DoC management is yet to be demonstrated clearly [23,24]. In this regard, patients responsiveness to VR training can be assessed by using EEG in terms of, e.g., event-related synchronization/desynchronization (ERS/ERD) and event-related spectral perturbation (ERSP), thus evaluating and regulating the specific effects of different VR protocols on cognition [25]. In fact, as interpreting a patient’s responsiveness to VR training remains challenging, especially in patients with DoC, an integration with EEG systems for recording brain activity could be useful to define a more specific stimulation protocol [26,27]. EEG could allow an objective quantification of the variations generated by the stimuli administered, could favor a greater customization of the sessions and help in verifying the progress of the rehabilitation program. For instance, EEG has proven capable of showing covert awareness within UWS samples by demonstrating high-level residual cognition, as the patients involved in an experiment regarding motor imagery were able to respond to commands [9,28,29]. Furthermore, a communication device can be achieved by conjugating EEG recording with cognitive treatments [30]. Last, evaluating EEG responsiveness to VR training may support the behavioral assessment to differentiate among patients with DoC. 

Consistent with these potential features of the EEG in the neurorehabilitation field, we designed a novel protocol to better manage patients with chronic DoC, whose possibility of recovery is often scarce.

We reported on the case of a young female patient with DoC who was provided with VR training to improve her cognitive-behavioral outcomes, which were monitored by means of both clinical and EEG data. We, then, carried out a systematic review of the literature to investigate the role of EEG and VR in the management of patients with DoC. 

## 2. Case Report

A 17-year-old woman experienced a car accident in December 2015. She was found in a coma state by the rescuers, was immediately intubated and transferred to the nearest emergency room. A brain computerized tomography scan revealed an extensive subarachnoid hemorrhage, midline structures shift, and multiple contusions mainly involving the left temporal and right parietal lobes. She was then admitted to an intensive care unit where she remained in coma for two weeks, after which she started to present with a low level of arousal with intermittent eye opening without any evidence of visual tracking or command-following. She had a tracheostomy and was fed through gastrostomy, and, about one month after the accident, she was admitted to a Special Unit of Permanent Welcome (SUAP), i.e., a long-term care ward that is dedicated to people in DoC whose rehabilitation and nursing needs are highly specific. Her neurologic examination showed only reflex responses (no voluntary movements), reduced bilateral blinking reflex, spastic tetraparesis, bladder and bowel incontinence. Her CRS-R score was 5 (auditory function: 1, visual function: 1, motor function: 1, verbal function: 1, communication: 0, and arousal: 1). She underwent intensive rehabilitation, which included sensory stimulation, physiotherapy and speech therapy. After about two years, the patient started to progressively show some inconsistent, fluctuant behavioral responses (eye blinking, weak lower limb adduction and right index movements, sporadic eye contact, poor visual anchoring), without intelligible verbalization, compatible with the possible evolution to MCS. Her CRS-R score was 7, the Disability Rating Scale (DRS) was 19, and the Rancho Los Amigos Levels of Cognitive Functioning (LCF) was 2. These values were intended as the baseline (T0). She was discharged at home, and, in outpatient regimen, she was provided with intensive personalized VR training using the Nirvana system (BTS Bioengineering; Garbagnate Milanese, Italy), to potentiate patient’s responses, especially the visual ones, with regard to anchoring and visual tracking. The patient came to our Robotic and Behavioral Neurorehabilitation Unit of the IRCCS Centro Neurolesi Bonino Pulejo—Piemonte (Messina, Italy), twice a week, to perform the treatment.

### 2.1. Intervention 

**Figure 1:**
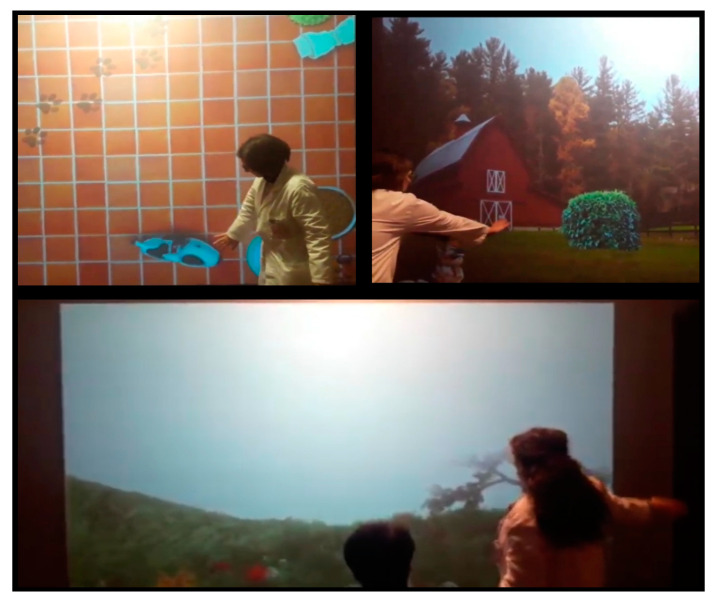
The Bts-Nirvana System. The therapist chooses the different scenarios according to the patient’s disability and needs; the patient may interact with the semi-immersive VR by standing or sitting (e.g., in a wheelchair, like our minimally conscious state (MCS) patient) in front of the interactive screen.

The training was conducted by a psychologist (M.G.M.) and a nurse (G.L.R.), in a VR environment using the Nirvana system. The latter is a semi-immersive system that promotes the recovery of impaired functions through exercises carried out in a non-invasive and realistic environment. The Nirvana system consists of a computerized software, two infrared sensors without markers, a video camera and a projector connected to a large screen (Figure 1). 

This device allows reproducing multiple virtual scenarios, with audiovisual feedback in response to movement, with greater awareness of performance and movements and total sensory involvement, and positive repercussions on the results of rehabilitation, as observed in various neurological diseases [31,32]. In addition, it allows adjusting the difficulty of the exercises in relation to the patient’s real skills and potential. The therapeutic sessions followed a specific protocol consisting of four phases. Initially, 10 min of relaxing screens were offered to prepare the patient for training. In the second phase (lasting about 15 min), we presented screens with familiar objects (animals, common objects), stimulating the patient’s eye contact and visual research. Thereafter, the therapists presented colored geometric figures and encouraged the patient to observe the stimuli (for about 15 min). Finally, a 10-min phase of cool-down relaxing screens was provided. The sessions were carried out twice a week. The screens presented to the patient were personalized and remained constant in the various phases. The experimental protocol lasted five months. We clinically assessed the patient at the twentieth, T20, and the last session, T40, and did an EEG recording during the VR training sessions (i.e., in a direct brain-to-VR interfacing) at the fourth, T4, twentieth, T20, and the last session, T40, to more objectively evaluate the patient’s responsiveness to VR training, so also to guide the rehabilitation protocol.

### 2.2. EEG Recording and Analysis

EEG was recorded using a standard 19-electrode headset wired to a BrainQuick system (Micromed; Mogliano Veneto, Treviso, Italy), at rest and during each VR trial. During the trial, the patient had to try to move the right index whether a pre-defined target was indicated by the therapist. Each trial consisted of four blocks of training in VR, each block consisting of 20 movement trials. Each movement trial consisted of a 10 s period of rest (baseline). Then, the patient was asked to try to perform right index extension whether a pre-defined target (familiar objects, colored geometric figures) was indicated by the therapist (every 7–15 s). The power spectral density (PSD) and the average event-related synchronization/desynchronization (ERS/ERD) [33,34,35] were calculated for the delta (1–3 Hz), theta (4–7 Hz), alpha (8–12 Hz), beta (13–30 Hz), and gamma (31–45 Hz) frequency bands and for F3, F4, Ce, C4, P3, and P4 electrodes. The 10 s baseline was adopted as a reference. Additionally, we calculated the time/frequency representation maps of ERS/ERD, i.e., the event-related spectral perturbation (ERSP), in the 8–30 Hz frequency range [36,37]. We focused on C3 and C4 electrodes as these were those showing the most significant and distinct activations (consistently with ERS/D data) and are the ideal candidate for assessing oscillatory activity within sensorimotor areas in relation to execution and imagery motor tasks [38]. Training performance was estimated as how many times ERD power exceeded the baseline measurement over central electrodes. We used the reliable change index (RCI) to determine whether there was a significant difference between the patient’s behavioral outputs at T20-T4 and T40-T4. We performed paired *t*-tests corrected for multiple comparisons to compare pre- and post-intervention means for each band on the continuous, dependent variable (ERS/ERD, ERSP, and training performance). Correlation analyses were carried out through Spearman correlation coefficient.

### 2.3. Outcomes

**Figure 2:**
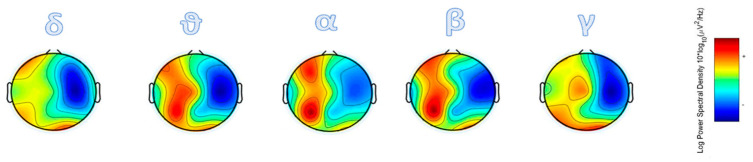
The resting state EEG (collected at T0), in which a large spectral peak in the θ band was found.

At baseline (T0), the resting state EEG showed a large, multiband spectral peak, prominently in the θ range (Figure 2). From a clinical point of view, already after the first sessions, we observed an improvement in the ocular fixation of the stimuli. Concerning the analysis of the EEG recorded during the task (conducted at T4), we found a whole-band ERD within all electrodes in the left hemisphere.

**Figure 3:**
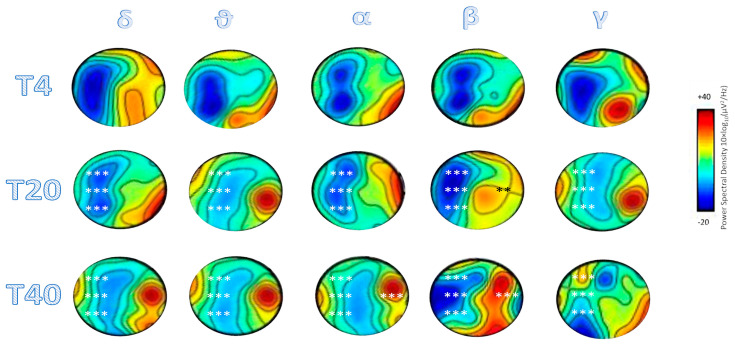
The scalp maps of event-related desynchronization/synchronization (ERD and ERS) across all channels. * refers to significant changes as compared to T4 (*** *p* < 0.001, ** *p* < 0.01).

The patient completed each session without any significant side effects (including vomiting, nystagmus, and pain). At T20, the patient maintained a stable eye contact with the stimuli presented, manifesting spontaneous eye-opening and directionality of the gaze. The patient achieved a CRS-R score of 9 (auditory function: 2, visual function: 2, motor function: 1, verbal function: 1, communication: 1, and arousal: 2) (T20 vs T4 RCI = −3.2), a DRS of 15 (RCI = 5.2), and a LCF of 2 (RCI non-significant). The whole band ERD within the left hemisphere electrodes, which was appreciable at T4, largely decreased in each electrode and frequency band (T20 vs T4 *t*-test, all *p* < 0.001) but beta band, where we found a further stronger ERD (all *p* < 0.001). Conversely, C4 showed a clear beta ERS (t_(2)_ = 18, *p* = 0.003) (Figure 3).

At T40, the patient presented with a CRS-R score of 13 (auditory function: 3, visual function: 4, motor function: 2, verbal function: 1, communication: 1, and arousal: 2) (T20 vs T4 RCI = −9.5), a DRS of 14 (RCI = 6.5), and a LCF of 3 (RCI = −2.2). The ERD reduction detected at T20 was still significant at the T40 vs T4 comparison within each left-hemisphere electrode and frequency range (all *p* < 0.001). However, beta frequency in C3 still showed a significant ERD (t_(2)_ = −38, *p* = 0.0007), as well as C4 showed a further stronger ERS in alpha and beta frequency ranges (both *p* < 0.001) (Figure 3).

**Figure 4:**
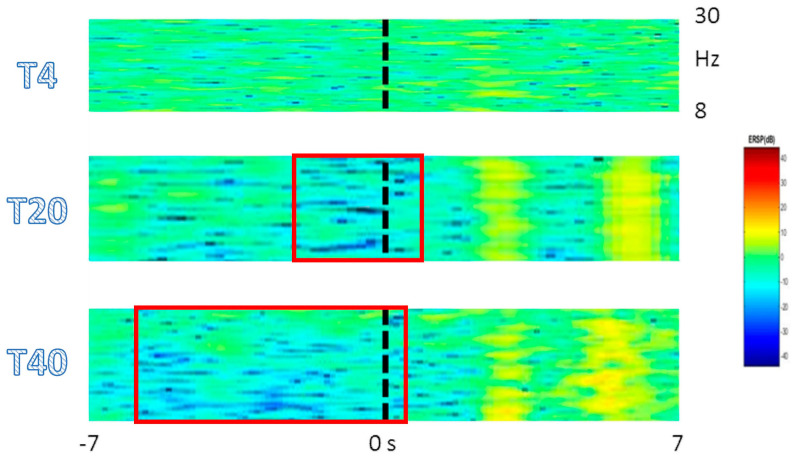
The extraction of event-related spectral perturbation (ERSP) maps from C3, which showed desynchronization between 8 and 30 Hz at T20 and T40 as compared to the baseline (T4). Significant time periods (all *p* = 0.01) are marked in red.

The extraction of ERSP maps as a time/frequency representation of ERD/ERS for all pre-post intervention trials showed a clear C3 desynchronization between 8 and 30 Hz at T20 and T40 as compared to T4 (both *p* = 0.01) (Figure 4).

Training performance (i.e., how many times ERD power exceeded the baseline measurement over central electrodes) increased from T4 (36 ± 2%) to T20 (62 ± 3%) (t_(3)_ = −7.1, *p* = 0.002) and from T4 to T40 (75 ± 10%) (t_(3)_ = −9.5, *p* = 0.001). Given that baseline motor impairment level may impact the ability to perform the VR task, we assessed the relationship between VR task performance, the resting-state alpha (that is related to cognitive and motor performance) [39], the beta ERD (that is related to motor performance, and the motor impairment (CRS-R). We found only a negative correlation between overall VR task performance (i.e., at T40) and CRS-R, which was not significant although (*r* = −0.842, *p* = 0.3).

## 3. Systematic Review

### 3.1. Search Strategy

The systematic review was performed using the PRISMA (Preferred Reporting Items for Systematic Reviews and Meta-Analyses) guidelines to investigate the role of EEG/VR in patients with DoC [40]. A comprehensive search of PubMed, Web of Science, Scopus and Google Scholar databases was performed, selecting the full-text articles. We combined the keywords and their synonyms in each database, as follows: (“Disorders of Consciousness” OR “Unresponsive wakefulness syndrome” OR “Minimally Conscious State”) AND (“virtual reality” OR “augmentative stimulation” OR “EEG” OR “Neurophysiological”). No filters were applied on the publication date of the articles and all the results of each database were included until March 2020. After the removal of the duplicates, all the articles were evaluated based on the titles and abstracts by three of the researchers, independently (D.L., A.M., and A.N.). These researchers read the full-text articles deemed suitable for the study and performed data collection to reduce the risk of bias (i.e., publication bias; time lag bias; language bias). In case of disagreement on the inclusion and exclusion criteria, the final decision was made by a fourth investigator (R.S.C.).

The inclusion criteria were: (i) patients with neurological signs or symptoms, with consciousness disorders; (ii) presence of a description or image of at least one EEG recording; (iii) dealt with virtual reality or augmented stimulation; (iv) English language; and (v) published in a peer-reviewed journal. The exclusion criteria were: (i) patients with medical or psychiatric comorbidities; (ii) animal studies; and (iii) conference proceedings, or reviews; (iv) alterations of consciousness other than the evolution of coma.

### 3.2. Data Extraction Process

**Figure 5:**
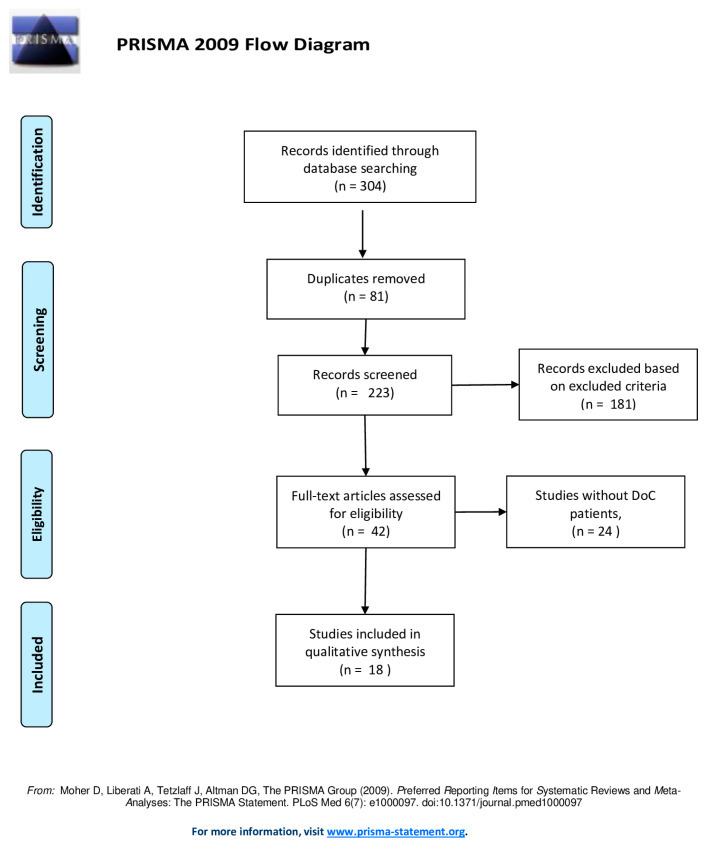
PRISMA flow-chart [41].

Data extraction was performed on 304 articles (Figure 5). We excluded: eighty-one articles as they were duplicates; one hundred eighty-one articles based on inclusion/exclusion criteria; twenty-four articles because they did not deal exclusively with DoC patients. The data were extracted on the basis of the following data: authors, year and type of publication (for example, conference proceedings, clinical case); characteristics of the participants involved in the study and purpose of the study; virtual reality training (yes or no); and presence of the EEG description (yes or no). After an accurate revision of full manuscripts, 18 articles satisfied the inclusion/exclusion criteria (Table 1). 

### 3.3. Results of the Systematic Review

The studies reviewed here used VR in either DoC assessment or rehabilitation, but they did not integrate EEG with VR. Indeed, different neurophysiological approaches, including EEG and event-related potentials (ERPs), have been used to evaluate patients’ outcomes or diagnosis. Specifically, (i) two studies used ERP to monitor residual cognitive functions [42,43]; (ii) eight studies used EEG/ERP to evaluate residual brain activity of DoC patients potentially suggesting cognition [44,45,46,47,48,49,55,56]; (iii) two studies used the enrichment environment or VR to stimulate cognitive function and awareness recovery in DoC patients, without EEG monitoring [13,14]; (iv) seven studies used other neurophysiological tools in DoCs, without EEG or VR, to evaluate residual brain activity of DoC patients [50,51,52,53,54,57]. These studies suggest that VR and several types of neurophysiological assessment, both individually and in combination, are potentially effective in corroborating DoC differential diagnosis and prognosis. However, some of the abovementioned studies suffer from several limitations, including a small sample, the lack of control group, a short follow-up, and the use of passive paradigms. Nonetheless, EEG data in combination with VR could be useful in evaluating the reactions induced in DoC patients, helping in the differential diagnosis. In this regard, in an attempt of bridging this gap, we used a combined EEG-VR approach in a patient with DoC, as described in the previous paragraphs.

## 4. Discussion

Two main issues arise from our case report and systematic review. First, VR could be effective to stimulate cognitive function and behavioral responsiveness of patients in MCS. Second, neurophysiological data, such as EEG and ERPs, in combination or not with VR, is proposed as a valuable tool to define the presence of patient responsiveness and differentiate the type of DoC.

Concerning the first point, our case-report, consistently with the literature data we reviewed here, suggests that VR could be a valuable methodology to support rehabilitation, as it allows obtaining positive functional motor and cognitive outcomes [13,14,48,56]. VR is the multisensory and interactive simulation of ecological scenarios, generally presented in a three-dimensional way, with which the patient can interact [15,58,59,60,61]. The VR devices use specific software with input-output peripherals that make the experience complex and engaging, promoting the improvement of the bodily functions of patients with motor/cognitive disabilities, as well as their well-being and participation [62,63,64]. VR allows being at the center of rehabilitation training, through two perceptive conceptions, i.e., immersion and presence [65]. “Immersion” is the objective perception of a sense of “sensory absorption” in the three-dimensional environment, whereas, “presence” is a subjective psychological state whereby the user is consciously involved in the virtual context. To stimulate our patient, we used the Nirvana system, which creates a VR semi-immersive environment, allowing to observe a three-dimensional scene projected on a screen: the playful environment could increase patient’s compliance, amplifying the effects of the rehabilitation treatment tailored to the actual needs of the patients [31,66,67]. Various studies have shown that VR activates different perceptual and experiential aspects, with the stimulation of multiple perceptual channels, thanks to the use of auditory and visual feedback [13,14,32,33]. This complex sensory stimulation may increase the patient’s awareness of his/her performance, inducing changes in neural plasticity processes [13,14,56,68,69,70]. These effects could be due both to the reactivation/amplification of brain neurotransmission and the involvement of mirror neurons, thanks to the visual-motor information coming from the observation of the stimuli in the VR screen [16]. Finally, VR can increase the level of excitement and awareness by stimulating the ascending reticular activation system, as suggested formerly [21,22]. Thus, VR could facilitate awareness of the environmental inputs, boosting neural plasticity to potentiate motor learning also in patients with DoC [23]. In line with this premise, we carried out multisensory stimulation in a semi-immersive VR environment using the Nirvana system on a patient with MCS, monitoring the cognitive-behavioural progresses using EEG. As far as we know, this is the first time that such a combined approach has been applied to patients with MCS. We found that the multisensory stimulation provided by means of the Nirvana system could enhance patient’s responses, especially visual ones (with particular regard to anchoring and visual tracking). Furthermore, our patient performed more intentional responses during the training, and this was objectively ascertained through the EEG monitoring. In particular, after the first sessions, we observed an improvement in the ocular fixation on stimuli; at T20, the patient was able to maintain stable eye contact when the stimuli were presented on the screen, manifesting spontaneous ocular opening and directionality of the gaze; and at T40, the patient presented spontaneous and purposeful responses to external stimuli. Our results, although obtained by a single case, could support the hypothesis that purposeful behavioral responsiveness of patients with DoC could be increased using VR training. Actually, the clinical scales highlighted an improvement in the global reactivity (as per CRS-R) and cognitive impairment (as per DRS). These data were paralleled by a retrain of sensorimotor oscillatory activity. Indeed, the EEG demonstrated a progressively stronger left sensorimotor beta ERD, which was also more specific (i.e., enhanced left sensorimotor beta-ERD, reduced whole/band left hemisphere ERD), and balanced by a right sensorimotor beta-ERS. Furthermore, a trial-by-trial more consistent responsiveness to the VR motor task, regardless of the presence of appreciable movements, was achieved.

According to previous studies, our data suggest that an intensive, repetitive, assisted, and task-oriented VR training could be able to retrain sensorimotor rhythms with overall positive effects on behavioral (CRS-R) and cognitive (DRS) outcomes in patients with MCS, in keeping with cognitive-motor learning principles [71]. In fact, a more intense and selective beta band entrainment with its interhemispheric counterbalance is a critical issue for cognitive and motor outcome improvement in neurorehabilitation settings [72]. Furthermore, we may hypothesize that the amount of sensorimotor information provided to the patient by means of VR training may have reshaped thalamo-cortical plasticity, which may explain the persistent remodulation of brain rhythms both within and outside sensorimotor areas and the large improvement of patient’s arousal and, partially, awareness [72,73]. In this regard, we have shown formerly that a motor training associated with VR is able to entrain several brain areas and their related brain rhythms (including the sensorimotor and, probably, those belonging to the mirror neuron system) involved in motor planning and learning in patients with stroke, thus leading to improved cognitive-motor performance [16].

Concerning the second point, the EEG data of our patient confirmed the diagnosis of MCS with certainty, as the patient showed an intentional modulation of her brain activity in response to VR training. This data was consistent with the literature evidence on the suitability of EEG-based approaches in understanding the processes subtending awareness and their pathophysiology [30,36,39,44,71,72,73]. In fact, some studies indicate that EEG, as well as other advanced neurophysiological approaches, could be useful to differentiate patients with DoC, with regard to those in a functional locked-in syndrome to reduce the still high misdiagnosis rate [46,47,49,56]. In particular, various types of neurophysiological data in a pre- and post-intervention design demonstrated that clinical neurophysiology could help in both assessing the clinical severity and measuring the brain activity related to the patient’s recovery [45,50,51,52,53,54,55,57]. Particularly, these studies employed neurofeedback techniques evaluated with EEG, EEG recording during motor imagery or cognitive tasks, neural network reconstruction and evaluation of cerebral blood flow during passive and active paradigms and in resting state modality, sleep parameter variability, cardiovascular and EEG responsiveness to nociceptive stimulation, and a bulk of biological responses (ERPs, EEG oscillatory activity, neural networks, autonomic responses) to visual and auditory stimuli (either artificial-using, e.g., transcranial currents- or ecological-using, e.g., BTS-NIRVANA), delivered individually or in combination [13,14,42,43,44,45,46,47,48,49,50,51,52,53,54,55,56,57]. All such approaches permit identifying even covert biological responses suggesting volitional cognitive activity and, thus, awareness as well as the potential to recover awareness. Interestingly, odd-ball paradigm visual or auditory ERPs permit ascertaining two main specific properties of aware cognitive processes: first, the demonstration of an ongoing, functional working memory, which means that the subject is likely aware as one has to be conscious of the provided mental representation (ERPs). Second, the identification of spatially and temporally distributed brain responses, which are unlikely when awareness is not present or not entrained [74]. Therefore, these indices may be useful to evaluate adequately motor-cognitive outcomes (with particular regard to anchoring and visual), and to monitor EEG responsiveness to a task (sensorimotor rhythms reshape) even in a VR environment, where it is suggested that the patient could be more stimulated [13,14,32,33]. In the current literature, two studies integrated VR with ERPs on patients with DoC by using the Neurowave (Khymeia SRL; Padua, Italy) a technologically advanced device that allows the automated programming and administration of sensory stimuli and the simultaneous monitoring of multiple biophysiological signals [42,43]. De Salvo et al. have carried out a pilot study on eleven UWS and five MCS individuals, observing that ERPs monitoring can be useful for assessing residual cognitive function, thus guiding the optimal management at individual level [42]. Another work by the same authors suggested that the ERP component could be a predictive marker for patients’ cognitive recovery. Furthermore, according to the authors, intensive sensorineural stimulation programs could help the recovery of cognitive and attentional functions in subacute cerebrovascular disease [43]. Indeed, Di Stefano et al. have observed that even in non-communicative disorders of consciousness, such as UWS and MCS, increasing the personal relevance and the complexity of stimuli seems to improve the activating effects on patients. In fact, they observed that enriched stimulation in an immersive environment could increase the number and/or goal-oriented quality of active movements in 12 patients with severe DoC in the post-acute phase [13]. Furthermore, Chen and Peng observed that a “coma stimulation program” that stimulates the five senses of the patients using VR can increase the patient’s responsiveness and improve the therapeutic process [14].

Last, brain responses evaluated using EEG/ERPs in a framework, or not, of VR may provide useful information concerning DoC prognosis. A preserved EEG/ERP responsiveness is an index of a potential to awareness recovery. However, the prognosis of a DoC patient remains not easily predictable, as it can vary from serious with negative exitus (death from bulbar suffering and cardiovascular collapse), to various degrees of recovery of consciousness (i.e., VS/UWS and MCS). In our previous work, we have observed that rehabilitation programs—which include physical therapy and multisensory stimulation (including tactile, visual and auditory) in addition to psychoactive drugs—can progressively promote awareness recovery [75].

## 5. Conclusions

As far as we know, this is the first time ever that EEG has been used during a VR training to more objectively monitor patients with DoC’s recovery. Such a combined approach has proven effective in defining the most appropriate stimulation protocol and promoting a better customization of the rehabilitation program. We are aware that findings from a single case report have many limitations, including epidemiological bias, impossibility of causal inference and generalization, and over-interpretation. Thus, our results should be confirmed by well-designed clinical studies. However, the data of our case-report are consistent with those from the studies we reviewed, showing that patient’s responses are not always easily interpretable using clinical scales only. By comparing previous studies regarding the use of other physiological indices and VR, we believe that our approach could be a valid tool to define DoC and reach the differential diagnosis, taking into account that around 40% of patients with UWS and MCS are misdiagnosed in clinical practice [76]. A quantitative assessment of command-following tasks (as per our combined EEG-VR approach) can be particularly helpful in dealing with differential diagnosis of these patients. In fact, this method can evaluate the specific behaviors of DoC patients, helping to validate both clinicians and caregivers’ observations. Indeed, the presence of relevant and objective data while assessing patients with Doc should be considered a fundamental starting point to better manage these frail and vulnerable individuals and their families. However, further randomized clinical trials are mandatory, as well as a meta-analysis of the literature, to be affirmative on the role of VR in patients with DoC, especially in combination with EEG.

## Figures and Tables

**Table 1:** The studies included in the systematic review. ES, experimental study; OS, observational study.

Author, Year	Study’s Design	Tools	Patients	Major Findings
De Salvo et al., 2015 [42]	ES	Neurowave system: Visual event-related potentials (ERPs)	11 vegetative state patients (VS) 5 Minimally Conscious State patients (MCS)	The authors found that ERPs monitoring can be useful for assessing residual cognitive function, thus guiding the optimal management at individual level.
De Salvo et al., 2019 [43]	ES	Neurowave system: Visual ERPs	18 ischemic subacute stroke patients	The authors showed that the P300 ERP component could be a predictive marker for cognitive recovery of ischemic subacute stroke patients. Intensive programs of neurosensory stimulation could facilitate recovery of cognitive and attentive functions in subacute cerebrovascular disease.
Keller et al., 2015 [44]	ES	Neurofeedback technique (NFB) evaluated with EEG	3 VS patients	The authors found that NFB can be used in patients in a state of insensitive insomnia.
Naro et al., 2018 [45]	OS	EEG	patients with chronic consciousness disorders (DoC)	The authors noted that EEG-based approaches can help differentiate patients with DoC.
Aricò et al., 2016 [46]	OS	EEG, including assessment of sleep structure and potential multimodal evoked recording, assessment of pain perception	14 DoC patients	The authors stress the presence of correlations between global brain connectivity, sleep structure and pain perception, which are related to the activity of the large thalamocortical and cortico-cortical networks underlying consciousness.
Naro et al., 2018 [47]	ES	EEG	14 patients with post-traumatic DoC 10 healthy controls	The authors found that mirror system evaluation can usefully be used to differentiate patients with DoC.
Cacciola et al., 2019 [48]	OS	EEG	45 DoC patients	The authors show that EEG-based approaches can help understand the processes that underlie consciousness and their pathophysiology.
Naro et al., 2017 [27]	ES	EEG Audiovisual Stimulation	10 healthy controls 7 MCS patients 9 UWS patients	The authors noted that the audiovisual stimulation paradigm can be used as a bed support tool to improve differential diagnosis in patients with DoC.
Naro et al., 2016 [26]	ES	EEG during a state of awake rest and performed a low-resolution electromagnetic brain tomography (LORETA)	DoC patient	The authors suggest that LORETA analysis may be useful in DoC differential diagnosis.
Hauger et al., 2017 [49]	ES	EEG ERP	14 patients with traumatic brain injury (TBI)	The authors found that ERP can index cognitive abilities early on after TBI and cognitive P300 can provide information on residual cognition and prognosis.
Di Stefano et al., 2012 [13]	ES	Virtual reality	20 DoC patients	The authors showed a difference in the patient’s response as a function of the stimulation context. A context with emotional richness and complexity of environmental stimuli can play a key role in evoking active behavior.
Chen and Peng, 2006 [14]	ES	Virtual reality	Coma patients	The authors suggest that a coma stimulation program that triggers the five senses of patients using VR can increase the patient’s responsiveness and improve the therapeutic process
Naro et al., 2018 [50]	OS	Changes in cerebral blood flow rate (CBFV) Functional transcranial Doppler (fTCD)	21 patients with DoC (10 patients with MCS and 11 with UWS) 25 healthy controls	The authors note that fTCD can be a quick and very simple tool for differentiating patients with MCS and UWS.
Leo, et al., 2016 [51]	OS	Ultra-late laser evoked potentials Skin reflex	12 MCS 10 UWS	The authors suggest that a broad spectrum electro-physiological assessment of autonomic nervous system functionality may support differential DoC diagnosis.
Keren et al., 1998 [52]	OS	ERPs	60 DoC patients	The authors observed that ERP recordings can help assess the severity of the injury and as a physiological index of brain activity related to recovery from DoC.
Fernández-Espejo et al., 2008 [53]	OS	Functional magnetic resonance imaging	3 VS patients 4 patients in MCS 19 healthy controls	The authors found that magnetic resonance imaging may be useful for identifying responses of brain activity, which can go unnoticed in a bed test.
Naro et al., 2017 [54]	ES	Repetitive transcranial magnetic stimulation	20 patients with DoC	The authors speculate that patients with UWS who demonstrate evidence of residual default mode network and external awareness network functional correlation may be misdiagnosed.
